# *F. prausnitzii* potentially modulates the association between citrus intake and depression

**DOI:** 10.1186/s40168-024-01961-3

**Published:** 2024-11-14

**Authors:** Chatpol Samuthpongtorn, Allison A. Chan, Wenjie Ma, Fenglei Wang, Long H. Nguyen, Dong D. Wang, Olivia I. Okereke, Curtis Huttenhower, Andrew T. Chan, Raaj S. Mehta

**Affiliations:** 1 Clinical and Translational Epidemiology Unit, Massachusetts General Hospital and Harvard Medical School, Boston, MA USA; 2 Division of Gastroenterology, Massachusetts General Hospital and Harvard Medical School, Boston, MA USA; 3 Department of Nutrition, Harvard T.H. Chan School of Public Health, Boston, MA USA; 4 Department of Psychiatry, Massachusetts General Hospital and Harvard Medical School, Boston, MA USA; 5 Department of Epidemiology, Harvard T.H. Chan School of Public Health, Boston, MA USA; 6 Channing Division of Network Medicine, Department of Medicine, Brigham and Women’s Hospital and Harvard Medical School, Boston, MA USA; 7 Department of Biostatistics, Harvard T.H. Chan School of Public Health, Boston, MA USA; 8 Department of Immunology and Infectious Diseases, Harvard T.H. Chan School of Public Health, Boston, MA USA; 9 Broad Institute of MIT and Harvard, Cambridge, MA USA

**Keywords:** Gut microbiome, Depression, Citrus fruits, Metagenomics, Metabolomics, Transcriptomics

## Abstract

**Background:**

The gut microbiome modulates the effects of diet on host health, but it remains unclear which specific foods and microbial features interact to influence risk of depression. To understand this interplay, we leveraged decades of dietary and depression data from a longitudinal cohort of women (*n* = 32,427), along with fecal metagenomics and plasma metabolomics from a substudy (*n* = 207) nested in this cohort, as well as an independent validation cohort of men (*n* = 307).

**Results:**

We report that citrus intake and its components are prospectively associated with a lower risk of depression and altered abundance of 15 gut microbial species, including enriched *Faecalibacterium prausnitzii*. In turn, we found a lower abundance of *F. prausnitzii* and its metabolic pathway, S-adenosyl-L-methionine (SAM) cycle I in participants with depression. To explore causality, we found that lower SAM production by *F. prausnitzii* may decrease intestinal monoamine oxidase A gene expression implicated in serotonin and dopamine synthesis.

**Conclusions:**

These data underscore the role of diet in the prevention of depression and offer a plausible explanation for how the intestinal microbiome modulates the influence of citrus on mental health.

Video Abstract

**Supplementary Information:**

The online version contains supplementary material available at 10.1186/s40168-024-01961-3.

## Introduction

Depression is a widespread and debilitating condition, affecting more than 280 million individuals worldwide [1]. The precise causes of depression are unknown [2], and treatment is often ineffective. Seventy percent of patients with depression fail to respond to initial treatment with antidepressant medications [3] and/or develop intolerable side effects to the drugs [4]. Therefore, identifying modifiable causes of depression and developing novel therapeutics are urgently needed.

Diet may be a promising avenue for depression prevention and management. Mediterranean-style diets have been associated with a nearly 35% reduced risk of depression [5–8], and Mediterranean diet has similarly shown reductions in mood symptoms [9]. Although the specific food groups that underlie these findings remain unclear, citrus, including oranges and grapefruits, have recently been linked with lower depression risk [10]. However, the mechanisms explaining diet-depression relationships remain unknown.

Apropos, considerable evidence suggests that the effects of diet on human health may be modulated by the microbiome [11, 12]. Increasing data also suggests that the gut microbiome plays a causal role in the development of mental illness via the “gut-brain axis,” a bidirectional communication between the central and enteric nervous system [13–15], plausibly via production of metabolites that travel to the brain [9], modulation of intestinal neurotransmitter production [16], and/or stimulation of the vagus nerve [17]. Despite these data, there have been no studies exploring how the gut microbiome may mechanistically influence diet-depression relationships.

To bridge these knowledge gaps, we analyzed the interplay between citrus consumption, the gut microbiome, and risk of depression, in over 32,427 participants. We prospectively examined the long-term intake of citrus in relation to depression and abundance of gut microbial taxa, and in turn, the associations of these species and their metabolic potential with depression. Integrating decades of detailed dietary and depression information, as well as metagenomics, metabolomics, metatranscriptomics, and host gene expression data, our study represents one of the first and most in-depth investigations of how the gut microbiome may influence the diet-depression axis.

## Materials and methods

### Study population

This cohort study received approval from the institutional review board (IRB) at both Brigham and Women's Hospital and the Harvard T.H. Chan School of Public Health (IRB protocol: Nurses' Health Study 2, number 2001P001128). Submission of a completed questionnaire was recognized by the IRB as implied informed consent. The study followed the STORMS checklist for microbiome research [18] and adhered to the STROBE guidelines for reporting observational studies.

A prospective study was carried out within the Nurses’ Health Study II (NHS2) (https://nurseshealthstudy.org/), an ongoing cohort study that initially recruited 116,429 female registered nurses in 1989 [19]. At enrollment and every 2 years afterwards, questionnaires were sent to participants to collect data on multiple lifestyle variables and medical history [20]. Diet was evaluated using validated semi-quantitative food frequency questionnaires (FFQs). FFQs were sent every 4 years since 1991 to participants. We defined baseline as 2003 when information on depression was first collected. After excluding those with missing dietary data and/or current or lifetime depression at baseline in 2003, there were 32,427 middle-aged women who were followed up until 2017.

A nested substudy within the NHS2, called the Mind Body Study, occurred over a 1-year period between 2013 and 2014. Women from the ongoing Nurses’ Health Study II (NHS2) were eligible to participate in the Mind Body Study if they met one of the following criteria: (a) they had taken part in the diet/physical activity validation study between 2011 and 2012 and were not involved in any other ongoing substudy, or (b) they had provided a second blood and urine sample between 2008 and 2011 and had completed the NHS2 2011 biennial questionnaire. 207 women between the ages of 55 and 65, who were oversampled for mental health issues including history of trauma/traumatic stress and depression, provided up to four stool samples and a fasting blood sample [21]. The Brigham and Women’s Hospital and Harvard T.H. Chan School of Public Health Institutional Review Boards approved the research protocol.

### Assessment of *citrus* intake

In NHS2, dietary consumption was evaluated using validated food frequency questionnaires (FFQs) collected every 4 years. Individuals were asked how often they consumed a standard serving of around 130 different items [22]. For citrus consumption, participants were asked how often (never to six or more servings per day) they consumed grapefruit, oranges, grapefruit juice, and orange juice over the preceding year. Total citrus intake was calculated by combining the consumption of each individual product. During follow-up, information was also gathered on other dietary parameters, including total energy, alcohol, vegetables, and other fruits as described elsewhere [23]. In our primary analysis, we used the most recent dietary variables to predict depression risk in the next 4 years in keeping with prior studies [24]. Of note, data from food frequency questionnaires collected in 2011—the most recent questionnaire—was used in association analysis with microbial features from the MBS (2013–2014). Diet quality was assessed by factor analysis, leading to a “healthy” or prudent dietary pattern, included as a covariate (see statistical analysis below) [25]. Additionally, micronutrient data, including vitamin C, folic acid, naringenin, and others, were collected every 4 years. Micronutrient intakes were computed by multiplying the frequency response by the nutrient content of the specified portion size, utilizing data from the US Department of Agriculture and manufacturers. For citrus consumption, participants were asked how often (never to six or more servings per day) they consumed grapefruit, oranges, grapefruit juice, and orange juice over the preceding year. Total citrus intake was calculated by combining the consumption of each individual food. During follow-up, information was also gathered on other dietary parameters, including total energy, alcohol, vegetables, and other fruits as described elsewhere [23]. In our primary analysis, we used the most recent dietary variables to predict depression risk in the next 4 years in keeping with prior studies [24]. Of note, data from food frequency questionnaires collected in 2011—the most recent questionnaire—was used in association analysis with microbial features from the MBS (2013–2014). Diet quality was assessed by through factor analysis, wherein citrus intake was excluded. This analysis yielded a “healthy” or prudent dietary pattern, which was subsequently included as a covariate (see statistical analysis below) [25]. Additionally, micronutrient data, including vitamin C, folic acid, naringenin, and others, were collected every 4 years. Micronutrient intakes were computed by multiplying the frequency response by the nutrient content of the specified portion size, utilizing data from the Harvard Food Composition Database (https://www.hsph.harvard.edu/nutrition-questionnaire-service-center/).

### Assessment of depression

In the larger NHS2 cohort, depression status was determined using self-reported history of physician/clinician-diagnosed depression and regular use of antidepressants. Every 2 years, participants were asked if they had ever been diagnosed with depression by a clinician. In addition, every 2 years, participants were asked about regular use of antidepressants, including selective serotonin reuptake inhibitors (SSRIs). For the purposes of our analysis, we did not include tricyclic antidepressants (TCAs) since previous data suggested that TCAs are commonly used for non-depression conditions, including chronic pain and neuropathy, and could misclassify many participants [26]. We then categorized depression by strict criteria (physician/clinician-diagnosis AND antidepressant use) or broad criteria (physician/clinician-diagnosis AND/OR antidepressant use), as in other studies [27]. To minimize misclassification, the strict definition was our primary outcome. In the MBS dataset, the same definition of depression as in the NHS2 study was utilized.

### Covariate assessment

Along with FFQs, all NHS2 participants completed biennial questionnaires reporting lifestyle and medical history, including incident of diabetes mellitus, hypertension, and dyslipidemia. In the MBS, participants also provided information on weight, medication use, and alcohol intake at the time of blood collection, indicating their weight, medication use, and alcohol intake. At the time of stool collection, participants completed a questionnaire capturing the Bristol stool scale and antibiotic use. Details of the stool collection have previously been provided [21]. MBS participants completed two validated semi-quantitative food frequency questionnaires [21] to record their food and beverage consumption over the prior 6 months at the beginning and end of the MBS.

### Blood collection and metabolomics profiling

The blood samples were MBS participants collected blood at home or at a clinic, and the blood samples were then transported overnight on ice. Plasma was isolated for liquid chromatography-tandem mass spectrometry (LC–MS/ MS) as previously described [28].

### Stool collection and metagenomic sequencing

In 2013, 207 participants provided up to four stool specimens at two 24–72-h intervals spaced 6 months apart, for a total of four samples per individual [21]. In brief, samples were collected at home and deposited into 5 mL of RNALater (Life Technologies), which was then transported by overnight service [21]. Library preparation and metagenomic sequencing were performed at the Broad Institute [29], following a method that has been previously described [21]. In brief, total nucleic acid was extracted from an aliquot of each stool sample. Utilizing a combination of chemical and mechanical lysis techniques, along with magnetic bead-based purification. Quantification of DNA samples was performed using a fluorescence-based PicoGreen assay. Next, we created Illumina sequencing libraries from 100 to 250 pg DNA. Before shotgun sequencing, libraries were combined by collecting equal volumes of each library from groups of 96 samples. Libraries were then subjected to sequencing (HiSeq2000 or 2500), resulting in approximately 10 million paired-end reads. The Picard suite (https://broadinstitute.github.io/picard) was used to perform post-sequencing de-multiplexing and generate BAM and FASTQ files. The bioBakery3 workflows were implemented for downstream processing [30]. In brief, KneadData deleted low-quality bases, reads, and human “contaminant” sequences. Then, MetaPhlAn3 was used to establish bacterial taxonomic profiles, which identify taxa to the strain level and quantify their relative abundances [31]. HUMAnN3 generated genomic functional profiles based on the pangenomes [32]. The relative abundance data underwent a preprocessing step in which features with no variance or more than 90% zeros were filtered out. Subsequently, an arc-sine square-root transformation was applied to mitigate the effects of zero inflation.

### Statistical analysis

#### Prospective association between citrus consumption and risk of depression

Person-years were accumulated from the date of return of the baseline questionnaire in the year 2003 to the date of depression diagnosis, death, loss to follow-up, or end of the follow-up period (1 June 2017), whichever happened first. Hazard ratios (HRs) and 95% confidence intervals (CIs) were computed using Cox proportional hazards models, with age stratification and adjustment for covariates including BMI, physical activity, smoking status, menopausal hormone therapy, total caloric intake, alcohol intake, comorbidities (history of diabetes mellitus, hypertension, dyslipidemia), social network level, sleep hours, and a prudent dietary pattern as a representative of overall dietary quality [33]. We performed this analysis using SAS software (version 9.4, Cary, NC). All statistical analyses were two-sided with a *P*-value less than 0.05 indicating statistical significance.

#### Prospective association of citrus consumption with overall microbiome communities and specific microbial species

To assess the association of citrus consumption with overall microbiome diversity, we used taxonomic data to generate Bray–Curtis distances at the species level conducting a repeated-measures PERMANOVA for taxa using 999 permutations (*adonis* function in the v4.1 package “vegan” 2.5–6) [34]. To avoid ordering-related issues, we independently calculated the total variance explained by each variable, as previously done [35]. The PCoA ordination in Fig. 2B was created using the cmdscale function of the R package “ggplot2” version 3.3.5. [36].

To investigate potential associations between citrus consumption and specific microbial species, we applied an arcsine square-root transformation to the taxonomic data, followed by a per-feature linear mixed-effects model (lme function, “nlme” package version 3.1–149 [37]) to fit the resulting abundances. Models were adjusted for age, BMI, caloric and alcohol intake, Bristol stool scale, antibiotic use, and diet quality. These covariates were selected as they may represent confounding variables, in terms of their potential impact on gut microbiome composition. All models included participant ID as a random effect.

To account for multiple hypothesis testing, nominal *p*-values were adjusted with a false discovery rate (FDR) target of 0.25. The circular phylogenetic tree in Fig. 2C was produced using GraPhlAn (Graphical Phylogenetic Analysis) [38], to illustrate the relationship between each microbial feature and overall citrus consumption (https://huttenhower.sph.harvard.edu/graphlan).

We employed Pearson correlation analysis to examine the associations between citrus intake and micronutrients and then used HAllA (http://huttenhower.sph.harvard.edu/halla) to associate the micronutrients primarily associated with citrus with the abundance of microbial species [39].

#### Association of specific microbial species with depression status

To investigate associations between specific microbial species and depression, we employed differential abundance testing to identify microbial taxa that exhibited significant differences in abundance between depressed and non-depressed participants. Using a generalized linear mix effect model with adjustment for the same covariates as above, we also examined the association between microbial features and depression status.

#### The association of microbial genes, enzymes, and pathways with depression status

Again, we used per-feature tests, to relate genes, enzymes, and pathways to depression status. To be included in downstream analyses, a taxonomic feature (UniRef90) or a pathway (MetaCyc) was required to have a minimum relative abundance of 0.01% in at least 10% of samples. Additionally, we filtered all enzyme commissions with a relative abundance of less than 0.001% in more than 10% of samples. To correct for multiple hypothesis testing, we controlled the false discovery rate (FDR) with a target rate of 0.05 for *q* values estimated from the per-feature tests.

#### Validation of gut microbial species on biomarker-related depression in Men’s Lifestyle Validation Study (MLVS)

We validated the association of gut microbial species and biomarker-related depression in an independent cohort, from the Men’s Lifestyle Validation Study (MLVS). The MLVS was a substudy embedded within the Health Professionals Follow-Up Study (HPFS) and comprised of 307 men; it is a parallel cohort to the Mind Body Study (MBS).

We investigated the relationship between microbial features and a neurotransmitter *z*-score, specifically the one with the highest area under the curve, which was the combination of serotonin and gamma-aminobutyric acid (GABA). We conducted the microbiome analyses with an FDR threshold of *q* < 0.25, after applying Benjamini–Hochberg correction. Analyses were performed using R version 4.0.1.

In exploratory analyses, consistent with previous work [11, 40, 41], we initially applied an FDR cutoff of 0.25 to allow for the detection of a broader range of potential associations, which is critical when working with high-dimensional data, such as microbial genes, enzymes, and pathways. This more relaxed threshold enabled us to detect more sensitively potential patterns and generate hypotheses by capturing a wider set of associations with depression status. However, given the large number of associations observed, we recognized the need to minimize the number of false positives in our confirmatory analyses. To ensure that the associations we report are robust and not merely due to chance, we applied a more stringent FDR threshold of 0.05 to downstream analyses, consistent with previous work [40], when specifically assessing the association of microbial features with depression. This stricter cutoff helps to increase the confidence in our findings, ensuring that only the most statistically significant and biologically meaningful associations are highlighted for further interpretation [42, 43].

### Study population

The HPFS is a long-term cohort study that began in 1986 and included 51,529 middle-aged to elderly male health professionals. Participants were followed every 2 years to gather information on their lifestyle, medical history, and health-related factors, with a high follow-up rate. The study utilized a sub-study within the HPFS called the MLVS, consisting of 908 men aged 65 to 80 without significant health conditions. In 2012–2013, 307 men were recruited for the MLVS and provided stool samples for analysis, with two sets of samples collected over a span of 6 months.

### Blood collection and metabolomics profile

As in the MBS, blood and fecal samples were collected among MLVS participants up to four time points, 6 months apart. The samples were stored in liquid nitrogen freezers after being immediately placed on ice and transported to the laboratory via overnight courier. Plasma metabolomics was performed using the same MBS platform with LC–MS/MS.

### Stool collection and metagenomic sequencing

Stool samples were stored in RNAlater fixative and the Illumina HiSeq sequencing platform was used for paired-end shotgun sequencing of the DNA and RNA samples [44]. Taxonomic profiles were obtained using the identical bioBakery3 workflow as in the MBS [31].

### Biomarker-based definition of depression

Based on prior literature suggesting that circulating neurotransmitters are linked with depression [45, 46], we generated a “depression score” using circulating levels of serotonin and GABA, which were integrated using a *z*-score. To evaluate the performance of this score we computed the Area under the receiver operating characteristic curve (AUC-ROC) and its corresponding 95% confidence interval (CI) in the MBS. Given good performance, we related microbial features to this score in the MLVS using the model below:

$$\text{Neurotransmitter}\;\text{z-score}\sim\;\text{microbial}\;\text{species+age+BMI+activity+alcohol intake+diet quality}+(1\vert \text{participant})$$

### Association between gut microbial RNA expression and host gene expression in the rectum

We investigated the association between gut microbial RNA expression and host gene expression by analyzing rectal biopsy samples from the Human Microbiome Project 2 cohort. This longitudinal cohort consisted of 132 individuals, including patients with Crohn’s disease, ulcerative colitis, and non-inflammatory bowel disease, intensely profiled over the course of 1 year. As described elsewhere [40], stool samples were profiled with metagenomics and metatranscriptomics. Rectal biopsies were also obtained during a baseline colonoscopy, with four to fourteen samples collected from each participant. All biopsies were kept at the collection site for a maximum of 2 months before being shipped overnight on dry ice for bulk RNAseq, byHiSeq 2000 or HiSeq 2500. Each run consisted of the paired-end sequencing of 101 base pairs with an index barcode read of eight base pairs, as described elsewhere.

### Statistical analysis

Host transcripts were selected a priori given their prior implications in brain-gut disorders and links to vagus nerve activity: tryptophan hydroxylase (*TPH1*) [47–49], serotonin transporter (*SLC6A4*) [47–49], monoamine oxidase A (*MAOA*) [47, 49], chromograffin A (*CHGA*) [47, 49], and serotonin receptor (*HTR4*) [49].

After applying arc-sine square-root transformation to both MTX and MGX datasets of MetaCyc pathways and using the “log2CPM” function in R to transform the raw count data from host transcriptomic gene into continuous, normally distributed data, the abundances were fitted with a per-feature linear mixed-effects model (see specification below) using the lme function from the R package; the Wald test was used to determine the significance of the association (FDR q 0.25). In turn, the models were adjusted for age, antibiotic use, and DNA copy number, which allows for biological and technical zero values while also controlling for underlying DNA levels as previous research has shown that RNA levels can be a function of the mere abundance of DNA [50].

$$Host\;gene\sim(intercept)\:+\:RNA\:+\:DNA\:+\:IBD\_diagnosis\:+\:(1\vert participant)$$

## Results

### Integration of long-term dietary, depression, and multi-omics data in an ongoing cohort study

To understand the role of the gut microbiome in modulating the association between citrus intake and depression, we first leveraged data from 32,427 middle-aged women from the ongoing cohort study, the Nurses’ Health Study II (NHS2, https://nurseshealthstudy.org/), which since 1989 has collected detailed dietary and health information every 4 years (Methods) [20]. Consistent with prior studies [10], to minimize misclassification of outcomes, depression was defined by self-report of both physician/clinician-diagnosis of depression and regular use of antidepressants [10]. Overall, women who ate more citrus were more likely to have a lower BMI, exercise more, and consume more calories/day compared to women who ate less citrus (Supplementary Table 1).

**Fig. 1:**
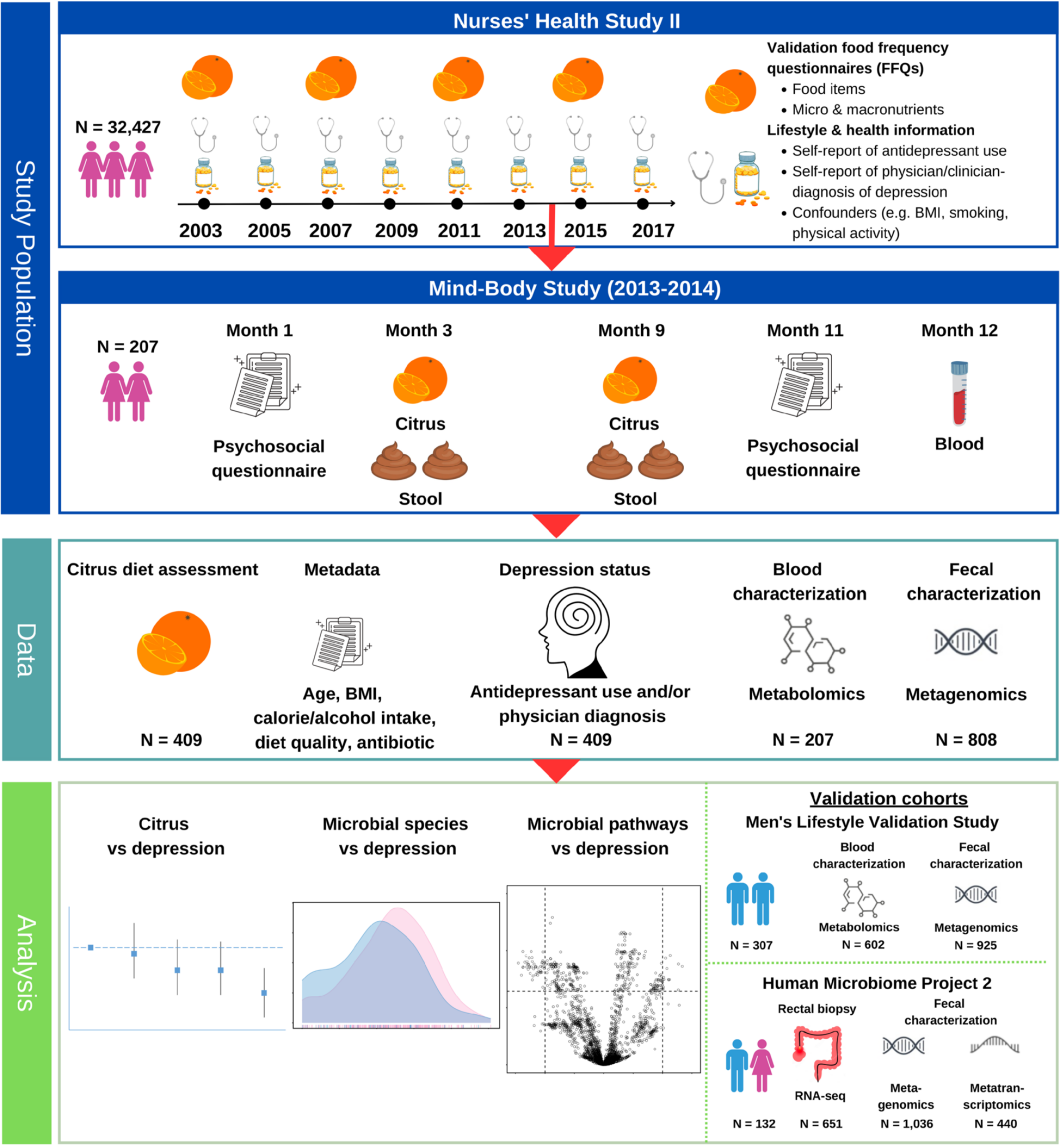
Identification of gut microbial species and metabolic pathways that may modulate relationships between citrus intake and depression. To understand the role of the gut microbiome in modulating the association between citrus intake and depression**,** we primarily leveraged data from the prospective Nurses’ Health Study II (NHS2), comprised of 32,427 middle-aged women, free of depression at baseline, who provided detailed depression, health, and dietary data every 2–4 years between 2003 and 2017. From 2013 to 2014, a nested sub-study of 207 women from the NHS2, the Mind Body Study (MBS), collected up to four stool samples, a fasting blood sample, dietary information, and detailed depression symptom questionnaires. Stool samples were then profiled through metagenomics (MGX), and blood was analyzed by untargeted metabolomics (MBX). In the analysis phase, we first prospectively assessed associations between citrus consumption with risk of depression and gut microbial species. Then, we related these species to depression status. Finally, we identified microbial metabolic pathways that could explain how microbial modulation of diet could plausibly influence mental health. Our findings were validated in the Men’s Lifestyle Validation Study (MLVS), a parallel cohort of men to the MBS

Within this longitudinal cohort, 207 women were selected to participate in the Mind Body Study (MBS), a year-long sub-study of postmenopausal women conducted between 2013 and 2014 that oversampled NHS2 women with mental health issues [21]. Participants provided up to four stool samples; a blood sample; and, akin to the parent cohort, they also completed two lifestyle, health, and dietary questionnaires, at the start and end of the study. In addition, they provided more detailed information about depression, through the completion of symptom scales, which were integrated with the criteria used to assess depression in the NHS2 (Fig. 1 and “Methods”). The baseline characteristics of participants in the MBS were similar to those in the larger parent NHS2 cohort (Supplementary Table 2).

### *Citrus* consumption is prospectively associated with decreased risk of depression

**Fig. 2:**
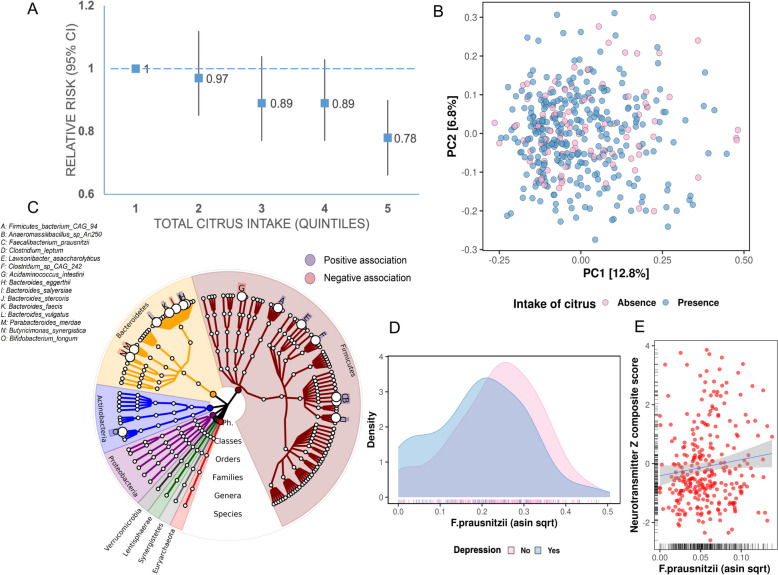
Citrus intake is inversely associated with depression, possibly via modulation of *F. prausnitzii*. **A** Greater intake of citrus is prospectively associated with a decreased risk of depression (*p*-trend 0.001) after adjustment for covariates including age, BMI, activity, smoking status, menopausal hormone therapy, total caloric intake, intake of alcohol, comorbidities (history of diabetes mellitus, hypertension, and dyslipidemia), social network level, median family income, overall diet quality, and sleep hours. **B** Global gut microbial taxonomic profiles vary according to the presence/absence of citrus intake (*R*.^2^ = 0.005, *P* = 0.001, adonis2). **C** Citrus consumption (assessed in 2011) is prospectively associated with 15 gut microbial species (assessed in 2013, linear mixed effects model, FDR *q* < 0.25). A circular cladogram of all species using GraPhlAn2[38] revealed that certain microbial species, such as *Faecalibacterium prausnitzii* and *Bifidobacterium longum*, showed an increase in abundance, while others, such as *Acidaminococcus intestini* and *Parabacteroides merdae*, exhibited a decrease. **D** Participants with depression have a lower relative abundance of *F. prausnitzii* compared to those without depression (linear mixed effects model, FDR *q* = 0.05, adjusted for age, BMI, calorie intake, antibiotics, alcohol, Bristol stool score, and diet quality. **E** In the independent MLVS cohort, greater abundance of *F. prausnitzii* was associated with a composite biomarker score for depression (*β* = 5.78, *p*-value 0.04)

From 2003 through 2017, we identified 2173 cases of depression among 32,427 women free of self-reported physician/clinician-diagnosed depression and regular use of antidepressants at baseline. Over 222,923 person-years of follow-up, compared to participants in the lowest quintile of citrus consumption, those in the highest quintile had a 22% lower risk of depression: HR 0.78 (95% CI 0.66–0.90), *p*-trend 0.001 (Fig. 2A), after multivariate (MV) adjustment for age, BMI, activity, smoking status, menopausal hormone therapy, total caloric intake, intake of alcohol, comorbidities (history of diabetes mellitus, hypertension, and dyslipidemia), social network level, median family income, overall diet quality, and sleep hours (Supplementary Table 3, “Methods”). To address the concern that depression occurrence may influence dietary behavior, we conducted a lagged analysis, adding a 4-year period between the assessment of dietary intake and each follow-up period. Associations were comparable: MV HR 0.80 (95% CI: 0.67–0.95, *p*-trend 0.002) (Supplementary Table 4). In another sensitivity analysis, similar to prior studies [10], we also considered a broader classification of depression, defined by self-reported regular use of antidepressants AND/OR physician/clinician-diagnosed depression, to identify 4950 cases of depression. Overall, the results were not meaningfully different: HR 0.83; 95% CI 0.75–0.91; *p*-trend 0.0002 (Supplementary Table 5).

To demonstrate specificity in the relationship between citrus intake and depression risk, we then investigated the associations between total fruit, total vegetable, apple, and banana intake with risk of depression. No significant associations were found (Supplementary Table 6), arguing against our findings being merely a proxy of healthy diet. Finally, to understand which components of citrus may protect against depression, we first correlated all available micronutrients (“Methods”) with citrus intake and subset those with modest or high correlation (*R* > 0.25), yielding biochanin A, formononetin, furocoumarin, naringenin, total flavanone, hesperidin, vitamin C without supplement, and luteolin (Supplementary Table 7). Among these, only naringenin and formononetin, two flavonoids found in citrus peels and expressed juice, were linked with depression (HR 0.79, 95% CI 0.69–0.91, HR 0.83, 95% CI 0.72–0.96, respectively, Supplementary Table 8). By contrast, vitamin C intake was not linked with depression.

### *Citrus* consumption is prospectively associated with abundance of gut microbial species

Next, we assessed the prospective relationship between citrus intake with abundance of gut microbial species. As described elsewhere, 808 fecal metagenomes were processed by the bioBakery3 suite [51], with taxonomic profiling by MetaPhlan3.0 [51] yielding a total of 144 microbial species across all participants after quality control (Fig. 1 and “Methods”). Citrus intake, assessed two prior years to stool collection, was linked with modest but significant effects on gut microbial community structures (Fig. 2B, R^2^ = 0.005, *P* = 0.001). In per-feature testing, using multivariate linear mixed effects models (“Methods”), we identified 15 microbial species that were associated with citrus intake (FDR *q* ≤ 0.25, Fig. 2C, Supplementary Table 9), with 11 showing a positive association and 4 showing a negative association. All models included age, BMI, total energy and alcohol intake, diet quality, and antibiotic use. Dietary quality was determined by empirical identification of a prudent dietary pattern, which was characterized by high consumption of vegetables, fruits, whole grains, and fish. As summarized elsewhere [52], food frequency questionnaire items were binned into roughly 40 food groups based on nutrient profiles or culinary usage, whereby alcoholic beverages were also included as a separate food group. Vitamin and mineral supplements were not included in the definition of the patterns. Factor analysis was then performed using an orthogonal rotation procedure to produce two maximally uncorrelated factors. Each participant was then assigned two factor scores, through the addition of reported frequencies of food group intakes, weighted by each factor’s loadings. These scores were then standardized using a *z*-score scale. We calculated the cumulative average of factor scores to capture long-term habitual consumption. As expected, citrus intake was positively associated with several abundant dietary fiber metabolizers and short-chain fatty acid producers, including *Faecalibacterium prausnitzii*,* Clostridium leptum*, and* Bifidobacterium longum* [53–55]. On the other hand, our findings revealed a negative correlation between citrus consumption and the presence of *Acidaminococcus intestini*, *Bacteroides stercoris*, and* Parabacteroides merdae*, which have been associated with chronic inflammatory disease, like inflammatory bowel disease [56].

We then investigated potential relationships between micronutrients derived from citrus intake and the 15 gut microbial species identified above. Notably, formononetin, furocoumarin, and naringenin showed a positive correlation with the abundance of *F. prausnitzii* (Supplementary Fig. 1, “Methods”). Moreover, biochanin A, formononetin, naringenin, hesperidin, and luteolin, which are subtypes of flavonoids, displayed negative associations with *Acidaminococcus intestini* and *Parabacteroides merdae* abundance. Interestingly, flavonoids have been proposed to modulate the diversity of the gut microbiome [57, 58], and prior studies have indicated a potential role of flavonoids in reducing depression [10]. Therefore, the presence of flavonoids, as essential microconstituents within citrus fruits, may exert influential effects on gut microbial communities.

### *Faecalibacterium prausnitzii,* modulated by citrus intake, is associated with lower risk of depression

We next investigated whether the 15 microbial species identified to be modulated by citrus intake were associated with depression in the MBS. Because of the unique phenotyping in this substudy, the definition of depression was made more rigorous to include recent symptoms in addition to clinician diagnosis of depression and antidepressant use (“Methods”). Using a generalized mixed effects model with multivariable adjustment (“Methods”), *F. prausnitzii* was lower in depressed versus non-depressed individuals (*β* = − 3.77, FDR *q* = 0.05) (Fig. 2D) after adjustment for age, BMI, calorie/alcohol intake, diet quality, and antibiotic use.

To validate these findings, we turned to a parallel cohort to the MBS, called the Men’s Lifestyle Validation Study (MLVS), comprised of 307 men. Citrus intake was similarly prospectively associated with a greater abundance of *F. prausnitzii* (*β* = 0.015, *p*-value = 0.04, Supplementary Fig. 2A). As a further validation step, we assessed whether *F. prausnitzii* appeared similarly protective efficacy against depression. Unlike the MBS, the MLVS cohort was not designed to collect detailed depression data. Therefore, we derived a composite biomarker score in both the MBS and the MLVS that summarized levels of serotonin and gamma-aminobutyric acid (GABA), which have been identified as potential metabolites for classifying the presence of depression in previous studies [45, 46, 59]. In both cohorts, participants provided a blood sample that underwent metabolomic profiling via liquid chromatography-tandem mass spectrometry (LC–MS) (“Methods”). In the MBS, this GABA/serotonin score demonstrated a superior discriminatory classification of depression vs. controls (AUC 0.86, 95% CI 0.75–0.97, “Methods”) (Supplementary Fig. 2B). Accordingly, using linear mixed effects models with multivariable adjustment (“Methods”), *F. prausnitzii* abundance in the MBS was associated with higher serotonin and GABA composite scores (*β* = 1.08, *p*-value = 0.027) (Supplementary Fig. 2C). Using the same linear mixed effect models in the MLVS, we subsequently found a significant positive association between *F. prausnitzii* abundance and higher composite serotonin and GABA scores in MLVS (*β* = 5.77, *p*-value = 0.04), lending further support to our earlier findings (Fig. 2E).

Taken together, these data show that citrus intake is prospectively associated with a lower risk of depression and an abundance of *F. prausnitzii* in two independent cohorts. In turn, the abundance of *F. prausnitzii* is inversely associated with depression in both cohorts.

### S-Adenosyl-L-methionine production by *F. prausnitzii* may influence gut-brain transmission

**Fig. 3:**
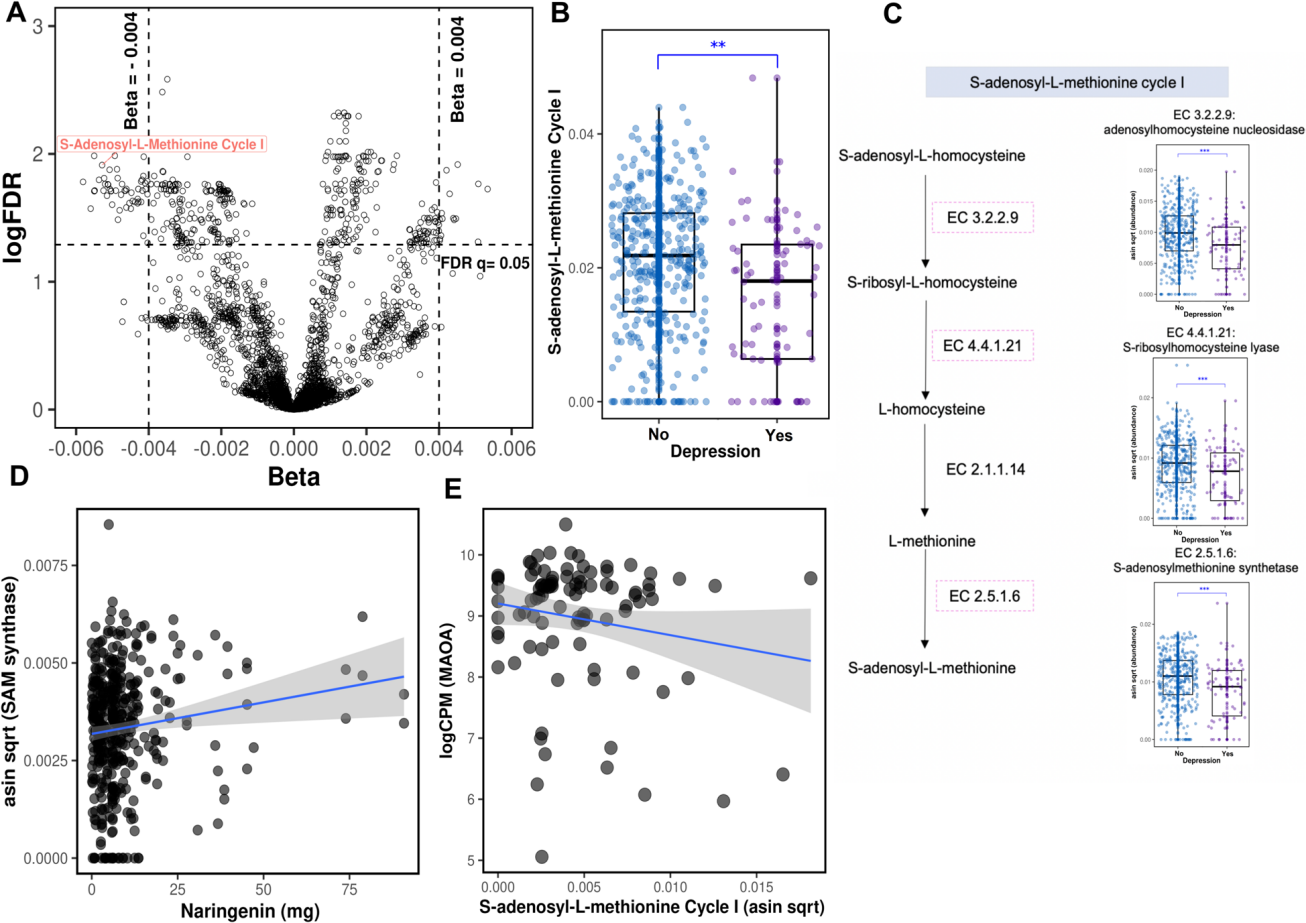
Metabolic pathways by which *F. prausnitzii* may influence depression. **A** Volcano plot demonstrating metagenomic carriage by 4098 pathways from all microbial species in relation to depression. S-Adenosyl-L-methionine cycle I pathway, encoded by *F. prausnitzii*, was among the strongest hits. **B** S-Adenosyl-L-methionine cycle I pathway of *F. prausnitzii* is reduced in depression (generalized linear mixed effects model, FDR *q* = 0.01). **C** In the SAM cycle pathway, S-adenosyl-L-homocysteine (SAH) is hydrolyzed to S-ribosyl-L-homocysteine by adenosylhomocysteine nucleosidase (EC 3.2.2.9) and then converted to L-homocysteine by S-ribosylhomocysteine lyase (EC 4.4.1.21). L-homocysteine is methylated to L-methionine using a methyl group from methylated folate, catalyzed by cobalamin-independent methionine synthase (EC 2.1.1.14). The SAM cycle is completed with the regeneration of SAM by SAM synthetase (EC 2.5.1.6). Abundance of the EC 3.2.2.9, EC 4.4.1.21, and EC 2.5.1.6 were significantly associated with depression (FDR q 0.004, 0.006, and 0.004, respectively). **D** Naringenin, a flavonoid specific to citrus fruit, was associated with an increased abundance of SAM synthetase, the rate-limiting step in SAM production (*β* = 0.00001, *p*-value = 0.038). **E** Greater abundance of S-adenosyl-L-methionine pathway was associated with lower monoamine oxidase A (*MAOA*) gene expression in the colon, which plays a role degradation of serotonin and dopamine

Having linked citrus intake with a greater abundance of *F. prausnitzii,* and having found that, in turn, *F. prausnitzii* was associated with a lower risk of depression, we next investigated the mechanisms by which this association may occur. Using linear mixed effects models with multivariable adjustment, we agnostically related all available (*n* = 4098) metabolic pathways stratified by their species contribution with depression in the MBS (Methods). Of these, we found 48 meeting our threshold (*q* < 0.05) that were contributed by *F. prausnitzii* (Supplementary Table 10). To minimize the odds of finding “passenger” functions, which merely correlate with an abundance of *F. prausnitzii*, we first took a data-driven approach and used prespecified criteria to filter out pathways with extremely high function-species abundance correlations (rho > 0.9, “Methods”). Then, we reviewed the remaining subset of 25 pathways and found that an abundance of the S-adenosyl-L-methionine (SAM) cycle I pathway was negatively associated with depression (*β* = − 0.005, *q* value 0.01, Fig. 3B). A growing body of evidence suggests that SAM supplementation in clinical trials lowers depression symptoms [60–63]. We then more deeply examined the enzymes involved in each step of the SAM cycle I pathway from MetaCyc. Consistent with the pathway analysis, we found a significant negative association between depression and adenosylhomocysteine nucleosidase (EC 3.2.2.9), S-ribosylhomocysteine lyase (EC 4.4.1.21), and S-adenosylmethionine synthetase (EC 2.5.1.6) (FDR *q* 0.004, 0.006, and 0.004, respectively) (Fig. 3C).

### Citrus and its micronutrients are also prospectively associated with a specific microbial gene in *F. prausnitzii*

To assess the association between citrus and its micronutrients with the SAM cycle I pathway in depression, we examined their relationship with the gene encoding the rate-limiting step [64], S-adenosylmethionine synthase (UniRef90_C7H250) in *F. prausnitzii.* Using multivariate linear mixed effects models, we observed a significant association between higher citrus intake and increased abundance of S-adenosylmethionine synthase (*β* = 0.0004, *p*-value 0.01, Supplementary Fig. 3). Additionally, we explored the potential impact of micronutrients linked to depression (above). Of these, naringenin was also linked with an increased abundance of UniRef90_C7H250 (*β* = 0.00001, *p*-value = 0.04, Fig. 3D). These findings support the notion that citrus and its micronutrients have the potential to influence the functional mechanisms of the SAM cycle I pathway, as mediated by the gene encoding S-adenosylmethionine synthase in *F. prausnitzii.*

### Expression of SAM cycle I is associated with lower intestinal serotonin and dopamine degradation activity in the *colon*

To explore how the metabolic activity of *F. prausnitzii* in the intestines may plausibly exert effects on brain health, we next assessed the association between gut microbial expression of the SAM cycle I pathway with host gene expression in the colon. Specifically, we leveraged data from a third independent cohort, the Human Microbiome Project 2 (ibdmdb.org, “Methods”), in which stool and rectal biopsies were collected from 132 participants with and without IBD. Based on prior literature identifying key genes regulating intestinal neurotransmitter production [47–49], we limited our exploratory analysis to *TPH1*,* SLC6A4*,* MAOA*,* CHGA*, and* HTR-4* (“Methods”). Greater microbial expression of the SAM I pathway by *F. prausnitzii*, and more broadly from all microbial species, was associated with lower *MAOA* expression (*β* = − 264.7, *p*-value 0.02 and *β* = − 75.6, *p*-value 0.02, respectively) (Supplementary Fig. 4, Fig. 3D). *MAOA* plays a crucial role in the degradation of neurotransmitters, including serotonin and dopamine, in the intestine [65]; thus, it may be that SAM production leads to greater neurotransmitter availability, which could then modulate vagus nerve activity [66].

## Discussion

For decades, diet has been linked with depression [5, 8, 67]. In parallel, the gut microbiome has been causally linked with depression and psychological distress phenotypes, specifically in animal models [13, 68–70]. Although the microbiome has been shown to mediate relationships between diet and cardiovascular disease [11], colorectal cancer [25], and irritable bowel syndrome (IBS) [71], no study to date has shown how diet may exert its effects on depression via the gut microbiome. Here, in two independent cohorts, we show that citrus intake is prospectively associated with a greater abundance of *F. prausnitzii*, which in turn, is associated with a lower risk of depression. This finding supports the notion that dietary interventions can mitigate or prevent depression symptoms, and, importantly, offers new avenues for therapeutic and/or biomarker development.

Notably, an abundance of *F. prausnitzii* has consistently been linked to human health, including metabolic syndrome, inflammatory bowel disease, and IBS [72–75]. Our data suggest that citrus consumption—even after adjusting for other healthy lifestyles and dietary behaviors—may preferentially select for the growth and activity of *F. prausnitzii*. More challenging is determining if *F. prausnitzii* causally lowers the risk of depression and how this might occur. Recent experimental data support that *F. prausnitzii* administration in mice ameliorates anxiety and depression symptoms. Furthermore, treatment with either *F. prausnitzii* or its supernatant raises intestinal serotonin levels in mice [76]. Thus, the SAM cycle I pathway offers a plausible mechanism by which *F. prausnitzii* may influence depression. SAM is a key methyl donor involved in the synthesis of mood- and behavior-regulating neurotransmitters, including serotonin and dopamine [77]. In support of this, SAM, when used as monotherapy or in conjunction with other antidepressants, has demonstrated encouraging and generally positive results in numerous clinical trials for major depressive disorder [60, 61, 63]. Given the vast diversity of *F. prausnitzii* subspecies, each carrying its own distinct enzymes, it remains to be explored if variation in depression across populations could be driven, in part, by strain-level heterogeneity.

More broadly, our work highlights the power of a multi-omics approach, leveraging nutritional epidemiology, metagenomics, metabolomics, metatranscriptomics, host gene expression, and clinical data to offer greater biological insight compared to prior studies that have primarily identified limited correlation between microbes and depression using 16S data [78]. Indeed, our multi-omic data allow us to speculate and generate hypotheses about how the biological processes in our gut may impact physiology in the brain in humans. Metabolites are key messenger molecules between microbes and the host, and while some may circulate to the brain [48], the vast majority are localized to the lumen [79] and/or are cleared by the liver [80]. Thus, interrogating how microbial metabolism corresponds with brain health demands interdisciplinary approaches.

We acknowledge that our study has several limitations. First, being observational, we cannot directly infer causal relationships, and the possibility of residual confounders remains. Nevertheless, we controlled for numerous variables including age, BMI exercise, and diet quality in our statistical analysis and found that antidepressant use did not significantly influence the results. In addition, the study participants were mostly white, middle-aged women, which could limit the generalizability of our findings to other populations and, importantly, may not capture the full diversity of microbial strains. Larger scale, international multi-omics studies could provide more definitive evidence here, including formal mediation or interaction analyses, and consideration of strain level data.

## Conclusions

Our study demonstrates a potential protective role of citrus fruit on the incidence of depression and suggests that *F. prausnitzii* and its metabolic activity may modulate the influence of citrus and its flavonoids on mood. Given the limitations of current pharmacologic treatment of depression [3, 4] our data support the potential of the gut microbiome in developing novel, molecularly informed biomarkers as well as modifying diet to prevent depression.

## Supplementary Information

Additional file 1: Table S1. Age-standardized characteristics in 2003 according to quintile of citrus intake. Table S2. Age-standardized characteristics at baseline of MBS according to quintile of citrus intake. Table S3. Quintiles of citrus consumption and risk of incident depression among women in the Nurses’ Health Study II. Table S4. 4-year lag analysis, showing the association between quintiles of citrus intake with risk of incident depression among women in the Nurses’ Health Study II. Table S5. Quintiles of the citrus consumption and risk of incident depression (broad definition) among women in the Nurses’ Health Study II. Table S6. Association between total fruit, total vegetable, apple, and banana intake with incident depression in the Nurses’ Health Study II. Table S7. Pearson correlation between citrus micronutrients with citrus intake (R > 0.25). Table S8. Association between Naringenin, and Formononetin intake with incident depression in the Nurses’ Health Study II. Table S9. The association between 15 microbial species, out of a total of 144, that were significantly linked to citrus intake. Table S10. 48 pathways, contributed to by F. prausnitzii were associated with depression risk ( q value < 0.05). Fig. S1. the associations between citrus-derived micronutrients and the abundance of gut microbial species using High-sensitivity pattern discovery in large, paired multi-omic datasets (HAllA). Fig. S2. (A) Greater citrus intake (assessed in 2010) was prospectively associated with F.prausnitzii abundance in MLVS (assessed in 2012–2013) (β = 0.015, p-value 0.04) (B) The composite score of circulating serotonin and GABA predicts depression status (AUC 0.86, 95%CI 0.75–0.97) (C) Greater abundance of F. prausnitzii was associated with our neurotransmitter composite z score in Mind–Body Study (β = 1.08, p-value 0.027). Fig. S3. Greater citrus intake was associated with increase abundance of a UniRef90_C7H250 (S-adenosylmethionine synthase)) (β = 0.0004, FDR q = 0.13). Fig. S4. Greater abundance of the S-Adenosyl-L-Methionine pathway, specifically encoded by F. prausnitzii, was associated with decreased expression of the MAOA gene in rectal enterochromaffin cells (β = -264.7, FDR q.

## Data Availability

All analysis code can be accessed at https://github.com/drraajmehta for purposes of reproducibility.
