# Supplementary material for: F. prausnitzii potentially modulates the association between citrus intake and depression
Source: Microbiome. 2024 Nov 14;12:237. doi: 10.1186/s40168-024-01961-3 (PMC11566247; doi:10.1186/s40168-024-01961-3)
Supplement: Supplementary file 2 — Additional file 1: Table S1. Age-standardized characteristics in 2003 according to quintile of citrus intake. Table S2. Age-standardized characteristics at baseline of MBS according to quintile of citrus intake. Table S3. Quintiles of citrus consumption and risk of incident depression among women in the Nurses’ Health Study II. Table S4. 4-year lag analysis, showing the association between quintiles of citrus intake with risk of incident depression among women in the Nurses’ Health Study II. Table S5. Quintiles of the citrus consumption and risk of incident depression (broad definition) among women in the Nurses’ Health Study II. Table S6. Association between total fruit, total vegetable, apple, and banana intake with incident depression in the Nurses’ Health Study II. Table S7. Pearson correlation between citrus micronutrients with citrus intake (R > 0.25). Table S8. Association between Naringenin, and Formononetin intake with incident depression in the Nurses’ Health Study II. Table S9. The association between 15 microbial species, out of a total of 144, that were significantly linked to citrus intake. Table S10. 48 pathways, contributed to by F. prausnitzii were associated with depression risk ( q value < 0.05). Fig. S1. the associations between citrus-derived micronutrients and the abundance of gut microbial species using High-sensitivity pattern discovery in large, paired multi-omic datasets (HAllA). Fig. S2. (A) Greater citrus intake (assessed in 2010) was prospectively associated with F.prausnitzii abundance in MLVS (assessed in 2012–2013) (β = 0.015, p-value 0.04) (B) The composite score of circulating serotonin and GABA predicts depression status (AUC 0.86, 95%CI 0.75–0.97) (C) Greater abundance of F. prausnitzii was associated with our neurotransmitter composite z score in Mind–Body Study (β = 1.08, p-value 0.027). Fig. S3. Greater citrus intake was associated with increase abundance of a UniRef90_C7H250 (S-adenosylmethionine synthase)) (β = 0.0004, FDR q [file 40168_2024_1961_MOESM1_ESM.docx]

|  | Frequency of citrus consumption (Quintiles) | | | | |
| --- | --- | --- | --- | --- | --- |
|  | QQ1  (< 0.5 svg/week)  N = 5717 | QQ2  (0.5-1.5 svg/week)  N = 9410 | QQ3  (1.5-2.5 svg/week)  N = 5077 | QQ4  (2.5-5.5 svg/week)  N = 6150 | QQ5  (> 5.5 svg/week)  N = 6073 |
| Age, years | 48.1 | 48.3 | 48.3 | 48.3 | 48.4 |
| Body mass index, kg/m^2^ | 26.5 | 26.4 | 26.4 | 26.0 | 25.3 |
| Smoking status, % |  |  |  |  |  |
| - Never smoking | 67.0 | 68.4 | 69.5 | 70.7 | 75.0 |
| - Current smoking | 7.9 | 6.4 | 5.6 | 5.0 | 4.0 |
| Physical activity, MET-hours/week^b^ | 20.3 | 21.2 | 21.7 | 23.4 | 24.9 |
| Current hormone therapy, % | 12.8 | 11.9 | 11.9 | 12.2 | 11.7 |
| Past hormone therapy, % | 10.1 | 9.5 | 9.0 | 9.2 | 8.8 |
| Total calories, kcal/d | 1686 | 1741 | 1877 | 1912 | 1994 |
| Alcohol, g/d | 5.2 | 5.7 | 5.8 | 5.7 | 5.6 |
| Average red meat intake, serving/week | 3.5 | 3.6 | 3.9 | 3.6 | 3.6 |
| Comorbidities, % |  |  |  |  |  |
| - Diabetes Mellitus | 2.3 | 2.3 | 1.8 | 1.9 | 1.3 |
| - Hypertension | 14.8 | 13.9 | 12.7 | 13.5 | 13.1 |
| - Dyslipidemia | 17.3 | 17.6 | 16.2 | 16.5 | 13.4 |
| Lowest social network, less than 2hrs/week of socializing, %^b^ | 60.5 | 60.1 | 58.1 | 57.5 | 56.9 |
| Lowest median income, less than $49k/year, %^b^ | 10.9 | 10.4 | 11.0 | 11.0 | 10.8 |
| Normal sleep hours, 7-8 hours/day, %^b^ | 68.0 | 70.3 | 70.9 | 69.2 | 71.0 |
| a All variables are age-standardized except age.  b Physical activity, social network levels, median family income, and sleep duration were reported from 2001. | | | | | |

**Supplementary table 1:** Age-standardized characteristics in 2003 according to quintile of citrus intake

**Supplementary table 2:** Age-standardized characteristics at baseline of MBS according to quintile of citrus intake

|  | Frequency of citrus consumption (Quintiles) | | | | |
| --- | --- | --- | --- | --- | --- |
|  | QQ1  (< 0.5 svg/week)  N = 5717 | QQ2  (0.5-1.5 svg/week)  N = 9410 | QQ3  (1.5-2.5 svg/week)  N = 5077 | QQ4  (2.5-5.5 svg/week)  N = 6150 | QQ5  (> 5.5 svg/week)  N = 6073 |
| Age, years | 56.3 | 56.5 | 56.5 | 56.5 | 56.5 |
| Body mass index, kg/m^2^ | 27.2 | 27.1 | 27.1 | 26.7 | 25.9 |
| Smoking status, % |  |  |  |  |  |
| - Never smoking | 67.0 | 68.1 | 69.3 | 70.5 | 74.8 |
| - Current smoking | 5.6 | 4.4 | 3.9 | 3.3 | 2.7 |
| Physical activity, MET-hours/week^b^ | 22.7 | 24.3 | 24.8 | 27.3 | 29.2 |
| Current hormone therapy, % | 14.4 | 13.6 | 14.3 | 14.0 | 14.0 |
| Past hormone therapy, % | 22.9 | 22.8 | 21.6 | 22.8 | 21.1 |
| Total calories, kcal/d | 1582 | 1624 | 1751 | 1805 | 1883 |
| Alcohol, g/d | 6.1 | 6.5 | 6.9 | 6.7 | 6.3 |
| Average red meat intake, serving/week | 2.9 | 3.4 | 3.6 | 3.4 | 3.2 |
| Comorbidities, % |  |  |  |  |  |
| - Diabetes Mellitus | 6.8 | 6.3 | 4.8 | 5.6 | 4.2 |
| - Hypertension | 33.1 | 31.8 | 30.7 | 30.7 | 29.3 |
| - Dyslipidemia | 46.3 | 46.9 | 44.1 | 44.2 | 40.7 |
| a All variables are age-standardized except age.  b Physical activity was reported from 2009. | | | | | |

**Supplementary table 3:** Quintiles of citrus consumption and risk of incident depression among women in the Nurses’ Health Study II

|  | **Strict Definition (n=2173)**  (Both Depression diagnosis by clinician and Use of Antidepressant) | | | | |  |
| --- | --- | --- | --- | --- | --- | --- |
|  | **Frequency of Consumption, No. of Servings** | | | | |  |
| Quintile* | QQ1 | QQ2 | QQ3 | QQ4 | QQ5 | p_trend_ |
| **Overall Citrus** |  |  |  |  |  |  |
| No. of cases | 319 | 608 | 363 | 453 | 430 |  |
| **Model 1:** Age-adjusted HR (95% CI) | 1 [reference] | 0.97  (0.85-1.11) | 0.89  (0.77-1.03) | 0.88  (0.76-1.01) | 0.75  (0.65-0.87) | 0.0003 |
| **Model 2:** Multivariate HR (95% CI) | 1 [reference] | 0.97  (0.85-1.11) | 0.89  (0.76-1.04) | 0.88  (0.76-1.02) | 0.77  (0.66-0.89) | 0.002 |
| **Model 3:** Multivariate HR (95% CI) with diet quality adjustment | 1 [reference] | 0.97  (0.85-1.12) | 0.89  (0.77-1.04) | 0.89  (0.77-1.03) | 0.78  (0.66-0.90) | 0.001 |
| **Model 4:** Multivariate HR (95% CI) with diet quality and fermented foods | 1 [reference] | 0.97  (0.85-1.12) | 0.89  (0.77-1.04) | 0.89  (0.77-1.03) | 0.78  (0.67-0.91) | 0.001 |
| Covariates: age; BMI; activity (metabolic equivalent tasks per week); smoking status (never, former, current); menopausal hormone therapy use (never, former, current); total caloric intake; intake of alcohol (g/d); comorbidity( history of diabetes mellitus, hypertension, dyslipidemia); social network level (<2hours/week, 3-10 hours/week, >11hours/week) ; prudent diet (quantiles); and sleep hours (≤6, 7-8, ≥9 hours/day); fermented foods (yogurt, oily fish, and nuts including peanuts and walnuts)  * quintiles: 1 (<0.5 serving/week), 2 (0.5-1.5 serving/week), 3 (1.5-2.5 serving/week), 4(2.5-5.5 serving/week), 5(>5.5 serving/week) | | | | | | |

**Supplementary Table 4:** 4-year lag analysis, showing the association between quintiles of citrus intake with risk of incident depression among women in the Nurses’ Health Study II

|  | **Strict Definition (n=2,027)**  (Depression diagnosis by clinician AND use of antidepressants) | | | | |  |
| --- | --- | --- | --- | --- | --- | --- |
|  | **Frequency of consumption, no. of servings** | | | | |  |
| Quantile* | QQ1 | QQ2 | QQ3 | QQ4 | QQ5 | p_trend_ |
| **Overall Citrus** |  |  |  |  |  |  |
| No. of cases | 276 | 571 | 367 | 441 | 372 |  |
| **Model 1:** Age-adjusted HR (95% CI) | 1 [reference] | 0.97  (0.84-1.12) | 0.92  (0.79-1.08) | 0.92  (0.79-1.07) | 0.74  (0.63-0.87) | <0.0001 |
| **Model 2:** Multivariate HR (95% CI)^1^ | 1 [reference] | 1.00  (0.86-1.16) | 0.94  (0.80-1.11) | 0.96  (0.82-1.13) | 0.80  (0.67-0.95) | 0.002 |
| **Model 3:** Multivariate HR (95% CI) with prudent diet adjustment | 1 [reference] | 1.00  (0.86-1.16) | 0.94  (0.80-1.11) | 0.96  (0.82-1.13) | 0.80  (0.67-0.95) | 0.002 |
| Covariates: age; BMI; activity (metabolic equivalent tasks per week); smoking status (never, former, current); menopausal hormone therapy use (never, former, current); total caloric intake; intake of alcohol (g/d); comorbidity( history of diabetes mellitus, hypertension, dyslipidemia); social network level (<2hours/week, 3-10 hours/week, >11hours/week) ; prudent diet (quantiles); and sleep hours (≤6, 7-8, ≥9 hours/day)  * quintiles: 1 (<0.5 serving/week), 2 (0.5-1.5 serving/week), 3 (1.5-2.5 serving/week), 4(2.5-5.5 serving/week), 5(>5.5 serving/week) | | | | | | |

**Supplementary table 5:** Quintiles of the citrus consumption and risk of incident depression (broad definition) among women in the Nurses’ Health Study II

|  | **Broad Definition (n=4950)**  (Depression diagnosis by clinician OR use of antidepressants) | | | | |  |
| --- | --- | --- | --- | --- | --- | --- |
|  | **Frequency of Consumption, No. of Servings** | | | | |  |
| Quintile* | QQ1 | QQ2 | QQ3 | QQ4 | QQ5 | p_trend_ |
| **Overall Citrus** |  |  |  |  |  |  |
| No. of cases | 775 | 1346 | 809 | 1028 | 992 |  |
| **Model 1:** Age-adjusted HR (95% CI) | 1 [reference] | 0.96  (0.88-1.05) | 0.90  (0.81-0.99) | 0.90  (0.82-0.99) | 0.80  (0.73-0.88) | <0.0001 |
| **Model 2:** Multivariate HR (95% CI)^1^ | 1 [reference] | 0.96  (0.88-1.05) | 0.90  (0.81-1.00) | 0.91  (0.82-1.00) | 0.82  (0.74-0.91) | 0.0005 |
| **Model 3:** Multivariate HR (95% CI) with diet quality adjustment | 1 [reference] | 0.96  (0.88-1.05) | 0.90  (0.82-1.00) | 0.91  (0.83-1.01) | 0.83  (0.75-0.91) | 0.0002 |
| Covariates: age; BMI; activity (metabolic equivalent tasks per week); smoking status (never, former, current); menopausal hormone therapy use (never, former, current); total caloric intake; intake of alcohol (g/d); comorbidity( history of diabetes mellitus, hypertension, dyslipidemia); social network level (<2hours/week, 3-10 hours/week, >11hours/week); prudent diet (quantiles); and sleep hours (≤6, 7-8, ≥9 hours/day) | | | | | | |

* quintiles: 1 (<0.5 serving/week), 2 (0.5-1.5 serving/week), 3 (1.5-2.5 serving/week), 4(2.5-5.5 serving/week), 5(>5.5 serving/week)

**Supplementary Table 6:** Association between total fruit, total vegetable, apple, and banana intake with incident depression in the Nurses’ Health Study II.

|  | **Strict Definition (n=2173)**  (Both Depression diagnosis by clinician and Use of Antidepressant) | | | | |  |
| --- | --- | --- | --- | --- | --- | --- |
|  | **Frequency of Consumption, No. of Servings** | | | | |  |
| Quintile | QQ1 | QQ2 | QQ3 | QQ4 | QQ5 | p_trend_ |
| **Fruits** |  |  |  |  |  |  |
| No. of cases | 483 | 523 | 434 | 388 | 345 |  |
| **Model 1:** Age-adjusted HR (95% CI) | 1 [reference] | 0.95  (0.84-1.07) | 0.93  (0.82-1.06) | 0.91  (0.80-1.05) | 0.93  (0.81-1.07) | 0.30 |
| **Model 2:** Multivariate HR (95% CI)^1^ | 1 [reference] | 0.98  (0.86-1.11) | 0.98  (0.85-1.11) | 0.97  (0.84-1.12) | 1.00  (0.86-1.16) | 0.98 |
| **Model 3:** Multivariate HR (95% CI) with prudent diet adjustment | 1 [reference] | 0.99  (0.87-1.13) | 1.01  (0.88-1.16) | 1.03  (0.89-1.20) | 1.09  (0.92-1.29) | 0.27 |
| - **Apple** |  |  |  |  |  |  |
| No. of cases | 327 | 597 | 456 | 559 | 234 |  |
| **Model 1:** Age-adjusted HR (95% CI) | 1 [reference] | 0.95  (0.83-1.09) | 0.87  (0.75-1.00) | 0.93  (0.81-1.06) | 0.85  (0.72-1.01) | 0.17 |
| **Model 2:** Multivariate HR (95% CI)^1^ | 1 [reference] | 0.97 (0.85-1.11) | 0.90  (0.78-1.04) | 0.97  (0.84-1.12) | 0.91  (0.76-1.09) | 0.55 |
| **Model 3:** Multivariate HR (95% CI) with prudent diet adjustment | 1 [reference] | 0.98  (0.85-1.12) | 0.92  (0.79-1.07) | 1.01  (0.87-1.17) | 0.97  (0.80-1.16) | 0.814 |
| - **Banana** |  |  |  |  |  |  |
| No. of cases | 345 | 549 | 465 | 587 | 227 |  |
| **Model 1:** Age-adjusted HR (95% CI) | 1 [reference] | 1.10  (0.96-1.27) | 1.02  (0.89-1.17) | 1.11  (1.00-1.27) | 0.99  (0.83-1.17) | 0.79 |
| **Model 2:** Multivariate HR (95% CI)^1^ | 1 [reference] | 1.12  (0.97-1.28) | 1.04  (0.90-1.19) | 1.15  (1.00-1.32) | 1.03  (0.87-1.22) | 0.75 |
| **Model 3:** Multivariate HR (95% CI) with prudent diet adjustment | 1 [reference] | 1.11  (0.97-1.27) | 1.04  (0.90-1.20) | 1.17  (1.02-1.34) | 1.06  (0.89-1.26) | 0.45 |
| **Vegetables** |  |  |  |  |  |  |
| No. of cases | 467 | 506 | 423 | 389 | 388 |  |
| **Model 1:** Age-adjusted HR (95% CI) | 1 [reference] | 1.00  (0.89-1.14) | 0.89  (0.78-1.02) | 0.88  (0.77-1.01) | 0.91  (0.79-1.04) | 0.054 |
| **Model 2:** Multivariate HR (95% CI)^1^ | 1 [reference] | 1.00  (0.88-1.13) | 0.89  (0.77-1.02) | 0.87  (0.75-1.00) | 0.88  (0.76-1.02) | 0.04 |
| **Model 3:** Multivariate HR (95% CI) with prudent diet adjustment | 1 [reference] | 1.00  (0.88-1.14) | 0.90  (0.78-1.04) | 0.89  (0.76-1.04) | 0.92  (0.77-1.11) | 0.28 |
| Covariates: age; BMI; activity (metabolic equivalent tasks per week); smoking status (never, former, current); menopausal hormone therapy use (never, former, current); total caloric intake; intake of alcohol (g/d); comorbidity( history of diabetes mellitus, hypertension, dyslipidemia); social network level (<2hours/week, 3-10 hours/week, >11hours/week) ; prudent diet (quantiles); and sleep hours (≤6, 7-8, ≥9 hours/day) | | | | | | |

| Citrus components | Correlation with Citrus intake (R) |
| --- | --- |
| Total flavanone | 0.77 |
| Biochanin A | 0.76 |
| Naringenin | 0.66 |
| Hesperidin | 0.65 |
| Vitamin C without supplement | 0.49 |
| Formononetin | 0.43 |
| Luteolin | 0.37 |
| Furocoumarin | 0.27 |

**Supplementary Table 7:** Pearson correlation between citrus micronutrients with citrus intake (R>0.25)

**Supplementary Table 8:** Association between Naringenin, and Formononetin intake with incident depression in the Nurses’ Health Study II.

|  | **Strict Definition (n=2173)**  (Both Depression diagnosis by clinician and Use of Antidepressant) | | | | |  |
| --- | --- | --- | --- | --- | --- | --- |
|  | **Frequency of Consumption, No. of Servings** | | | | |  |
| Quintile | QQ1 | QQ2 | QQ3 | QQ4 | QQ5 | p_trend_ |
| **Naringenin** |  |  |  |  |  |  |
| **Model 1:** Age-adjusted HR (95% CI) | 1 [reference] | 0.89  (0.78-1.02) | 0.87  (0.76-0.99) | 0.78  (0.68-0.90) | 0.74  (0.64-0.85) | <0.0001 |
| **Model 2:** Multivariate HR (95% CI)^1^ | 1 [reference] | 0.90  (0.78-1.02) | 0.89  (0.78-1.01) | 0.81  (0.70-0.93) | 0.78  (0.68-0.90) | 0.0005 |
| **Model 3:** Multivariate HR (95% CI) with prudent diet adjustment | 1 [reference] | 0.90  (0.79-1.03) | 0.89  (0.78-1.02) | 0.82  (0.71-0.94) | 0.79  (0.69-0.91) | 0.001 |
| **Formononetin** |  |  |  |  |  |  |
| **Model 1:** Age-adjusted HR (95% CI) | 1 [reference] | 1.06  (0.95-1.19) | 0.96  (0.84-1.11) | 0.97  (0.85-1.10) | 0.80  (0.69-0.92) | 0.001 |
| **Model 2:** Multivariate HR (95% CI)^1^ | 1 [reference] | 1.06  (0.94-1.18) | 0.96  (0.83-1.10) | 0.99  (0.86-1.12) | 0.83  (0.72-0.96) | 0.01 |
| **Model 3:** Multivariate HR (95% CI) with prudent diet adjustment | 1 [reference] | 1.05  (0.94-1.18) | 0.96  (0.84-1.11) | 0.99  (0.86-1.13) | 0.83  (0.72-0.96) | 0.009 |
| Covariates: age; BMI; activity (metabolic equivalent tasks per week); smoking status (never, former, current); menopausal hormone therapy use (never, former, current); total caloric intake; intake of alcohol (g/d); comorbidity( history of diabetes mellitus, hypertension, dyslipidemia); social network level (<2hours/week, 3-10 hours/week, >11hours/week); prudent diet (quantiles); and sleep hours (≤6, 7-8, ≥9 hours/day) | | | | | | |

**Supplementary Table 9:** The association between 15 microbial species, out of a total of 144, that were significantly linked to citrus intake.

| Species | Beta | FDR q value |
| --- | --- | --- |
| *Firmicutes_bacterium_CAG_94* | 0.00691607 | <0.0001 |
| *Bacteroides_faecis* | 0.02484706 | 0.00072035 |
| *Anaeromassilibacillus_sp_An250* | 0.00444001 | 0.00484936 |
| *Lawsonibacter_asaccharolyticus* | 0.00962369 | 0.01024869 |
| *Bacteroides_vulgatus* | 0.04761032 | 0.0420821 |
| *Clostridium_sp_CAG_242* | 0.00497964 | 0.06047345 |
| *Acidaminococcus_intestini* | -0.0144585 | 0.08314794 |
| *Bacteroides_eggerthii* | 0.02813545 | 0.13560862 |
| *Bacteroides_salyersiae* | 0.00954252 | 0.15644457 |
| *Clostridium_leptum* | 0.00612089 | 0.15644457 |
| *Bacteroides_stercoris* | -0.0396148 | 0.15644457 |
| *Bifidobacterium_longum* | 0.01609665 | 0.17657055 |
| *Faecalibacterium_prausnitzii* | 0.0266938 | 0.17657055 |
| *Parabacteroides_merdae* | -0.0161925 | 0.24832803 |
| *Butyricimonas_synergistica* | -0.003358 | 0.24832803 |

**Supplementary Table 10:** 48 pathways, contributed to by *F. prausnitzii* were associated with depression risk ( q value <0.05)

| Pathway names | β | FDR q value |
| --- | --- | --- |
| PWY.724..superpathway.of.L.lysine..L.threonine.and.L.methionine.biosynthesis.II. | -0.0049 | 0.010 |
| PWY.5097..L.lysine.biosynthesis.VI. | -0.0055 | 0.010 |
| THRESYN.PWY..superpathway.of.L.threonine.biosynthesis. | -0.0041 | 0.011 |
| PWY.6151..S.adenosyl.L.methionine.cycle.I. | -0.0053 | 0.012 |
| PWY.6305..putrescine.biosynthesis.IV. | -0.0049 | 0.013 |
| HISTSYN.PWY..L.histidine.biosynthesis. | -0.0051 | 0.013 |
| PWY.6124..inosine.5.phosphate.biosynthesis.II. | -0.0049 | 0.015 |
| COA.PWY..coenzyme.A.biosynthesis.I. | -0.0052 | 0.015 |
| PWY.4242..pantothenate.and.coenzyme.A.biosynthesis.III. | -0.0054 | 0.016 |
| GLCMANNANAUT.PWY..superpathway.of.N.acetylglucosamine..N.acetylmannosamine.and.N.acetylneuraminate.degradation. | -0.0042 | 0.016 |
| PWY.5686..UMP.biosynthesis. | -0.0058 | 0.017 |
| PWY.6123..inosine.5.phosphate.biosynthesis.I. | -0.0047 | 0.017 |
| GLYCOGENSYNTH.PWY..glycogen.biosynthesis.I..from.ADP.D.Glucose. | -0.0052 | 0.017 |
| PWY.5667..CDP.diacylglycerol.biosynthesis.I. | -0.0051 | 0.017 |
| PWY0.1319..CDP.diacylglycerol.biosynthesis.II. | -0.0051 | 0.017 |
| DTDPRHAMSYN.PWY..dTDP.L.rhamnose.biosynthesis.I. | -0.0051 | 0.017 |
| PEPTIDOGLYCANSYN.PWY..peptidoglycan.biosynthesis.I..meso.diaminopimelate.containing. | -0.0051 | 0.019 |
| PWY.6507..4.deoxy.L.threo.hex.4.enopyranuronate.degradation. | -0.0046 | 0.019 |
| PWY.6700..queuosine.biosynthesis. | -0.0041 | 0.019 |
| PWY.7357..thiamin.formation.from.pyrithiamine.and.oxythiamine..yeast.. | -0.0055 | 0.019 |
| TRNA.CHARGING.PWY..tRNA.charging. | -0.0047 | 0.019 |
| GALACTUROCAT.PWY..D.galacturonate.degradation.I. | -0.0046 | 0.019 |
| PWY.7111..pyruvate.fermentation.to.isobutanol..engineered.. | -0.0049 | 0.019 |
| PWY.1042..glycolysis.IV..plant.cytosol.. | -0.0052 | 0.019 |
| COA.PWY.1..coenzyme.A.biosynthesis.II..mammalian.. | -0.0050 | 0.019 |
| VALSYN.PWY..L.valine.biosynthesis. | -0.0050 | 0.020 |
| ARGSYN.PWY..L.arginine.biosynthesis.I..via.L.ornithine.. | -0.0055 | 0.020 |
| PWY.7400..L.arginine.biosynthesis.IV..archaebacteria.. | -0.0055 | 0.020 |
| PWY.7221..guanosine.ribonucleotides.de.novo.biosynthesis. | -0.0045 | 0.020 |
| GLUTORN.PWY..L.ornithine.biosynthesis. | -0.0048 | 0.021 |
| PWY.7219..adenosine.ribonucleotides.de.novo.biosynthesis. | -0.0051 | 0.021 |
| PWY.6121..5.aminoimidazole.ribonucleotide.biosynthesis.I. | -0.0043 | 0.023 |
| PWY.6387..UDP.N.acetylmuramoyl.pentapeptide.biosynthesis.I..meso.diaminopimelate.containing. | -0.0044 | 0.023 |
| PWY.7242..D.fructuronate.degradation. | -0.0043 | 0.024 |
| PWY.6527..stachyose.degradation. | -0.0031 | 0.024 |
| PWY.6317..galactose.degradation.I..Leloir.pathway.. | -0.0044 | 0.024 |
| PWY.6386..UDP.N.acetylmuramoyl.pentapeptide.biosynthesis.II..lysine.containing. | -0.0045 | 0.024 |
| PWY.6122..5.aminoimidazole.ribonucleotide.biosynthesis.II. | -0.0043 | 0.024 |
| PWY.6277..superpathway.of.5.aminoimidazole.ribonucleotide.biosynthesis. | -0.0043 | 0.024 |
| ARGSYNBSUB.PWY..L.arginine.biosynthesis.II..acetyl.cycle. | -0.0050 | 0.025 |
| PWY.5177..glutaryl.CoA.degradation. | -0.0046 | 0.026 |
| PWY.6737..starch.degradation.V. | -0.0056 | 0.027 |
| PWY.6609..adenine.and.adenosine.salvage.III. | -0.0045 | 0.027 |
| PWY.6897..thiamin.salvage.II. | -0.0048 | 0.028 |
| PWY66.422..D.galactose.degradation.V..Leloir.pathway. | -0.0039 | 0.033 |
| PWY0.1296..purine.ribonucleosides.degradation. | -0.0048 | 0.033 |
| THISYNARA.PWY..superpathway.of.thiamin.diphosphate.biosynthesis.III..eukaryotes. | -0.0038 | 0.041 |
| PWY.5695..urate.biosynthesis.inosine.5.phosphate.degradation. | -0.0037 | 0.046 |

**
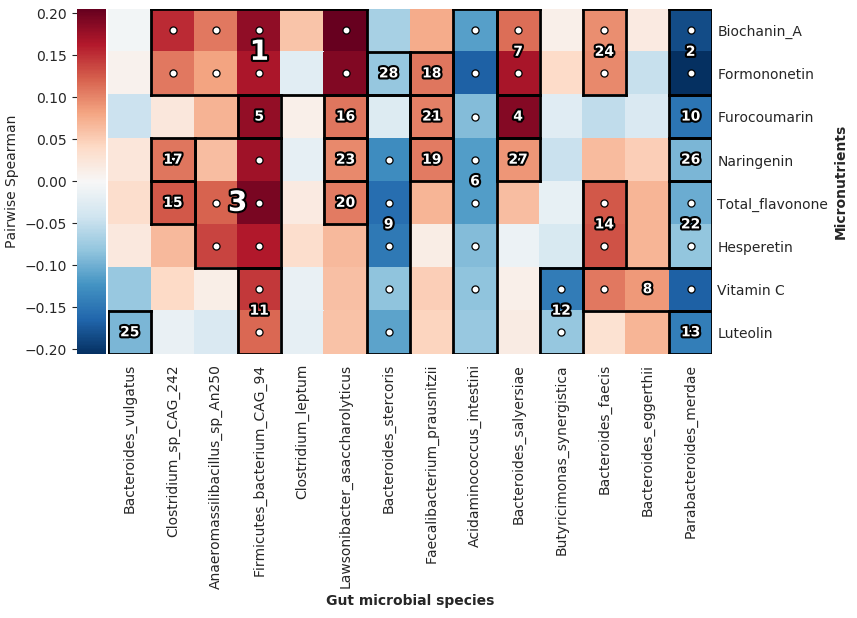
**

**Supplementary figure 1:** the associations between citrus-derived micronutrients and the abundance of gut microbial species using H**igh-sensitivity pattern discovery in large, paired multi-omic datasets**(HAllA)


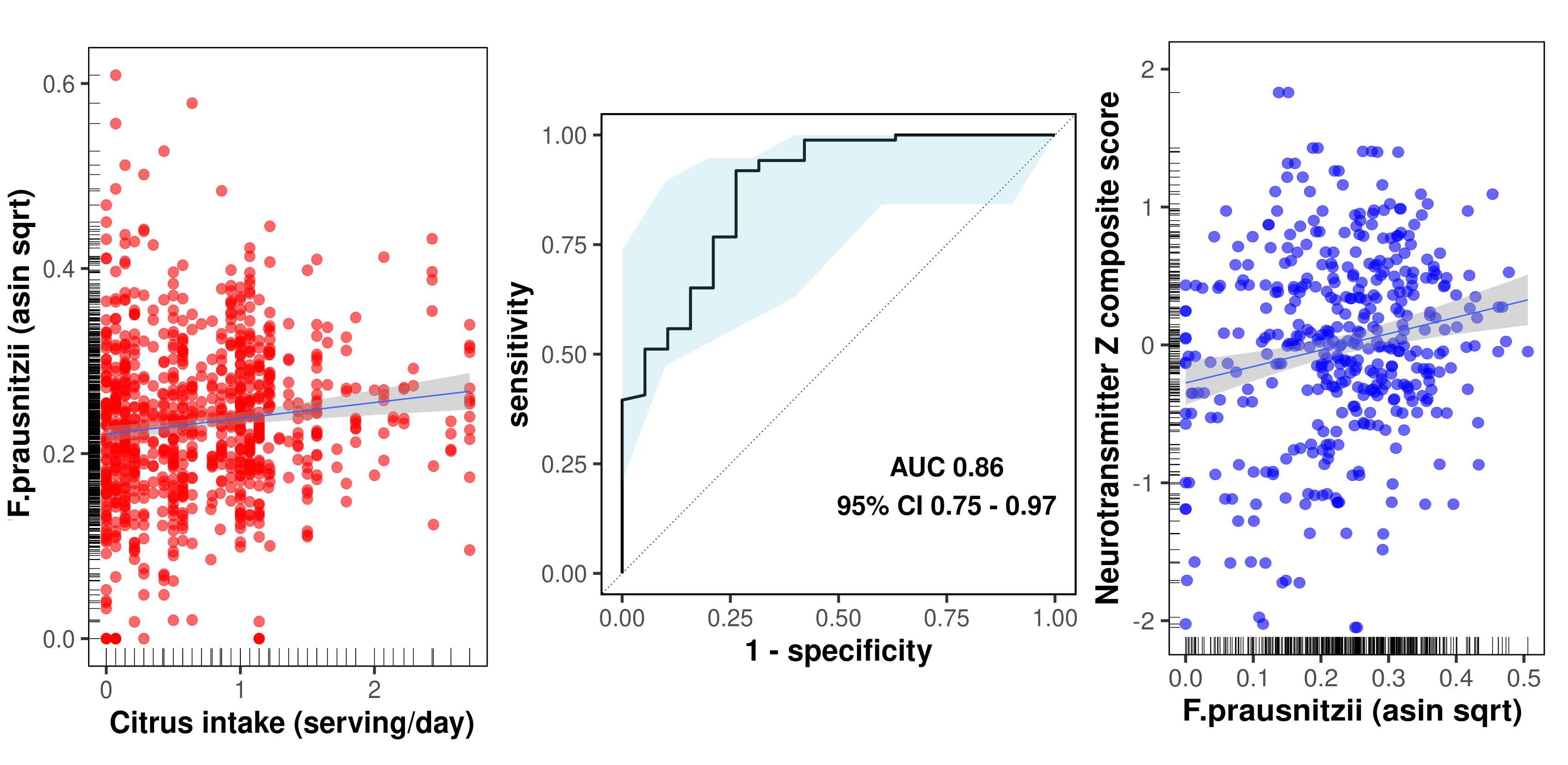


**Supplementary figure 2: (A)** Greater citrus intake (assessed in 2010) was prospectively associated with *F.prausnitzii* abundance in MLVS (assessed in 2012-2013) (β= 0.015, p-value 0.04) (**B**) The composite score of circulating serotonin and GABA predicts depression status (AUC 0.86, 95%CI 0.75-0.97) (**C**) Greater abundance of *F. prausnitzii* was associated with our neurotransmitter composite z score in Mind-Body Study (β = 1.08, p-value 0.027)


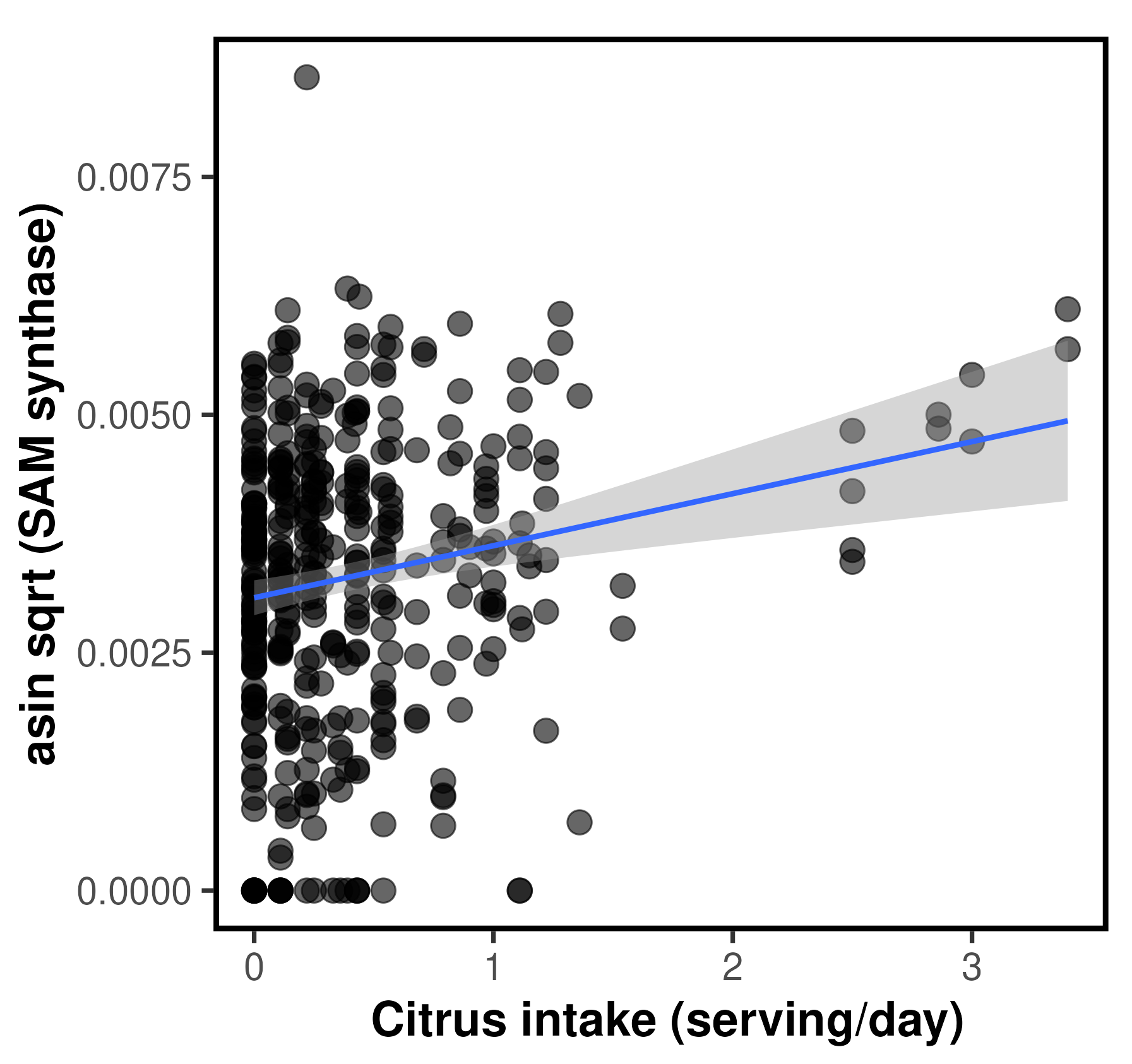


**Supplementary figure 3:** Greater citrus intake was associated with increase abundance of a UniRef90_C7H250 (S-adenosylmethionine synthase)) (β = 0.0004, FDR q =0.13)*.*


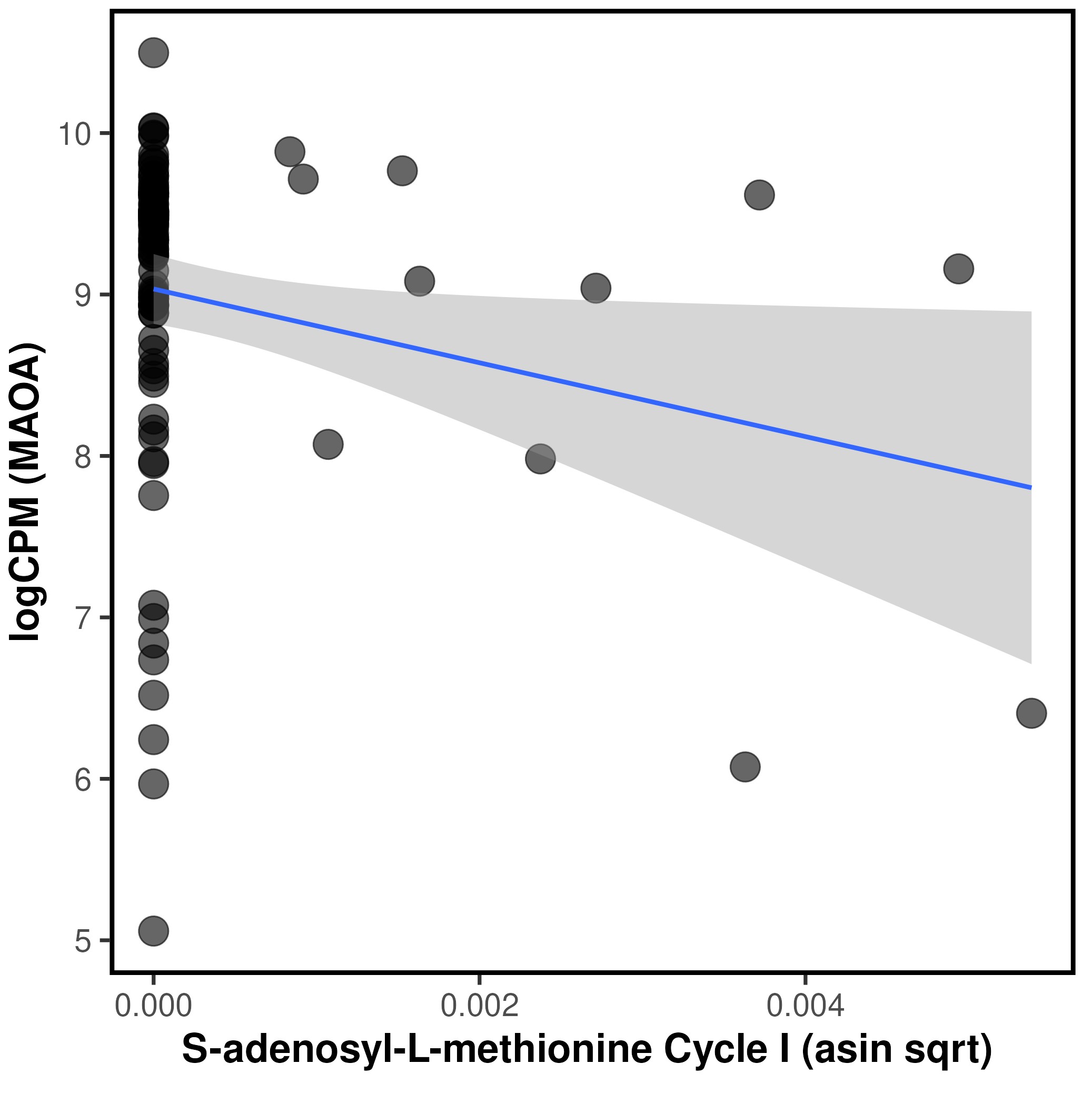


**Supplementary figure 4:** Greater abundance of the S-Adenosyl-L-Methionine pathway, specifically encoded by *F. prausnitzii*, was associated with decreased expression of the *MAOA* gene in rectal enterochromaffin cells (β = -264.7, p-value 0.02)
